# Common mental health problems experienced by adolescents living with HIV in sub-Saharan Africa: A scoping review

**DOI:** 10.1371/journal.pgph.0006832

**Published:** 2026-10-23

**Authors:** Celumusa Ndlangamandla, Musa Mamba

**Affiliations:** 1 Johns Hopkins School of Nursing, Johns Hopkins University, Baltimore, United States of America; 2 Eswatini Ministry of Health, Mbabane, Eswatini; London School of Hygiene and Tropical Medicine, UNITED KINGDOM OF GREAT BRITAIN AND NORTHERN IRELAND

## Abstract

Adolescents living with HIV (ALWH) carry a substantial burden of mental health problems, yet summary data remain limited. Previous reviews, particularly systematic reviews, have focused mainly on diagnosed disorders among adults or broader populations of young people living with HIV, while substance use and emotional and behavioural symptoms remain understudied and often underreported. This scoping review synthesized existing evidence on common mental health problems among ALWH aged 10–19 years in sub-Saharan Africa (SSA). We searched five electronic databases (PubMed, PsycINFO, CINAHL, Embase, and Open Access for Africa) and Google Scholar, and screened the bibliographic references of selected articles for additional studies. Search terms were combined using Boolean operators and organized into four concept categories: HIV, age, mental health, and region. Eligible studies were original, peer-reviewed research published in English between 2020 and 2026, conducted in SSA, and reporting on mental health among ALWH aged 10–19 years. The search yielded 427 records, of which 29 duplicates were removed. Of the 398 articles screened, 317 were excluded at the abstract stage and a further 57 after full-text review, leaving 24 articles for inclusion. The review identified six common mental health problems among ALWH in SSA: depressive disorders and symptoms, generalized anxiety, suicidality, emotional and behavioural problems, posttraumatic stress, and substance use problems. Depression and suicidality were the most frequently reported, whereas substance use, generalized anxiety, posttraumatic stress, and emotional and behavioural problems were less frequently reported. ALWH in SSA experience a range of mental health problems that may affect their well-being and engagement in HIV care. Routine mental health screening, prevention, and psychosocial support should be integrated into HIV care for this population. Strengthening integrated mental health and HIV services may help address unmet needs and improve health outcomes among ALWH in SSA.

## Introduction

The number of adolescents living with HIV (ALWH) continues to increase worldwide [1,2]. Globally, an estimated 2.21 million adolescents aged 10–19 years were living with HIV in 2025, of whom 84% resided in sub-Saharan Africa (SSA) [2–4]. A growing body of evidence indicates that ALWH are at increased risk of experiencing mental health problems and disorders [4,5]. Globally, approximately one in seven adolescents experiences a mental health problem, which accounts for around 15% of the total disease burden among individuals aged 10–19 years [6]. In Africa, an estimated 37 million adolescents were living with a diagnosed mental health condition in 2019 [7]. Despite this substantial burden, mental health problems among adolescents frequently remain undiagnosed and untreated [7]. Untreated mental health conditions during adolescence can impair psychosocial development and limit individuals’ ability to lead productive and fulfilling lives, with broader implications for societal and national development [7].

Adolescents account for approximately 23% of the population in SSA and play a critical role in the social and economic development of their families and communities, representing the next generation of the workforce and leadership [7,8]. However, ALWH, particularly those in SSA, face substantial challenges that may undermine their health and future potential [8–11]. These challenges include limited access to healthcare, HIV-related stigma and discrimination, fear of disclosing HIV status to family members, orphanhood, caregiving responsibilities, poverty, and the ongoing impact of the HIV epidemic [8,10]. The challenges associated with living with HIV may be particularly consequential during adolescence, a developmental period characterized by substantial physical, social, and cognitive changes as young people establish their identities and transition toward adulthood [12]. Adolescents also navigate evolving peer relationships and social connections, which can increase vulnerability to risky health behaviours and mental health problems [13,14]. Although adolescence can be challenging in its own right, these challenges may be compounded among ALWH by having an incurable, potentially fatal, and frequently stigmatized virus [14]. Consequently, ALWH may experience substantial mental health challenges [15].

The mental health needs of ALWH may be further exacerbated by limitations within healthcare systems. Fragmented healthcare systems in which mental health and HIV services are not integrated can contribute to undiagnosed and untreated mental health problems [16,17]. Moreover, limited mental health training and specialization among healthcare providers (HCPs) caring for ALWH [18,19] may contribute to underdiagnosis and unmet mental health needs [20]. Although evidence on the extent of mental health problems among adolescents in SSA remains limited [12], the interrelated social, economic, developmental, and healthcare-related challenges experienced by ALWH suggest that these problems may be widespread [21]. Given that SSA has the world’s fastest-growing adolescent population, there is an increasing urgency to ensure that ALWH receive appropriate mental health care and support [22].

Several initiatives have been implemented to support ALWH, although these efforts have primarily focused on improving HIV treatment and clinical outcomes rather than directly addressing mental health [23]. These initiatives include Teen Clubs, adolescent-friendly HIV services, and expert clients [24]. Teen Clubs are psychosocial peer-support groups for ALWH that provide education on HIV and its treatment while fostering a sense of community [25]. Through shared experiences, adolescents can support one another, strengthen coping mechanisms, combat stigma, and build self-confidence, potentially contributing to improved clinical and mental health outcomes [25,26]. Adolescent-friendly HIV services aim to improve access to HIV testing, prevention, and age-appropriate care that is acceptable to young people, ultimately supporting treatment adherence and health outcomes [27,28]. Expert clients are lay healthcare workers who have voluntarily disclosed their HIV-positive status and serve as role models for ALWH. They provide peer support and contribute to improved treatment adherence, retention in care, and youth-friendly service delivery [29]. Although these approaches may provide important secondary benefits for psychosocial well-being, their primary goal remains the improvement of clinical HIV outcomes [23]. Thus, despite existing efforts to support ALWH, their mental health needs in SSA remain largely unmet [16].

Significant progress has been achieved in HIV prevention and treatment [16], culminating in the United Nations’ ambitious goal of ending AIDS as a public health threat by 2030 [30]. Achieving this goal requires greater attention to the mental health needs of ALWH [31]. Unaddressed mental health problems may interfere with HIV prevention and treatment behaviours and contribute to ongoing HIV transmission [31]. Moreover, adolescents play a critical role in shaping HIV prevention behaviours and influencing perceptions of HIV risk and prevention [6,31]. Acquired immunodeficiency syndrome (AIDS) continues to be the number one cause of illness and death among ALWH in sub-Saharan Africa [3]. Addressing mental health during adolescence is therefore important not only for individual well-being but also for sustaining progress in the HIV response [16].

Despite a growing body of primary studies and systematic reviews examining mental health disorders among ALWH, limited scoping reviews have consolidated the broader spectrum of common mental health problems experienced by this population. Existing evidence remains fragmented and largely focused on discrete psychiatric diagnoses, limiting its usefulness for understanding the breadth of mental health needs and informing comprehensive screening and care. In particular, substance use problems and emotional and behavioural symptoms have received limited attention and are often underreported in the literature. This omission may obscure important dimensions of the mental health burden experienced by ALWH and overlook factors that may contribute to suboptimal health outcomes. Consequently, there is limited clarity regarding the range, patterns, and interrelated nature of common mental health problems experienced by ALWH in SSA. A comprehensive synthesis is therefore needed to map the existing evidence and identify gaps in knowledge across the spectrum of mental health problems affecting this population. Accordingly, this scoping review aimed to synthesize existing evidence on common mental health problems experienced by ALWH aged 10–19 years in SSA, informed by descriptive content analysis.

## Materials and methods

### Search strategy and database selection

The authors conducted a systematic and comprehensive literature search of five electronic databases (PubMed, PsycINFO, CINAHL, Embase, and Open Access for Africa), along with Google Scholar to identify relevant literature. The five electronic databases were selected to provide complementary coverage of the biomedical, psychological, behavioural, nursing, allied health, and African health literature relevant to mental health among ALWH. This combination of databases was intended to maximize the breadth and relevance of the evidence identified for the review.

The following search terms in combination with Boolean operators were used to identify relevant articles: (“HIV” OR “HIV infections” OR “HIV/AIDS” OR “HIV-1/HIV-2” OR “human immunodeficiency virus” OR “acquired immunodeficiency” OR “HIV-positive” OR “acquired immune deficiency syndrome” OR “AIDS” OR “acquired immunodeficiency”) AND (“adolescent” OR “adolescen*” OR “juvenile” OR “teenage*” OR “teen*” OR “youth*”) AND (“mental disorders” OR “mental health problems” OR “mental health issues” OR “mental health disorder*” OR “mental illness” OR “mental disability” OR “mental health condition” OR “mental distress” OR “mental instability” OR “psychosis” OR “psychological disorders” OR “psychiatric conditions”) AND (“sub-Saharan Africa” OR “Africa South of the Sahara” OR “Southern Africa” OR “Central Africa” OR “Western Africa” OR “Eastern Africa”). Medical Subject Headings (MeSH) terms were used in PubMed, while Emtree terms were used in Embase. Additionally, manual screening of the reference lists of included articles identified eight additional eligible studies, which were imported into Covidence and processed alongside records retrieved from the database search. The last literature search was conducted on 7 June 2026.

### Inclusion and exclusion criteria

Eligible studies were original, peer-reviewed research published in English between 2020 and 2026. Studies had to be conducted in sub-Saharan Africa and focus on mental health among ALWH aged 10–19 years. The publication period was restricted to 2020–2026 to capture recent evidence reflecting contemporary HIV treatment, adolescent-friendly healthcare, and mental health service contexts, while providing an updated synthesis of the literature on mental health among ALWH in SSA. Review articles and other non-original research were excluded.

### Search results

All identified articles were uploaded into Covidence, a web-based platform that supports systematic and scoping reviews, including study screening, data extraction, and risk-of-bias assessment [32,33]. Duplicate articles were automatically identified and removed, followed by manual verification. The literature search yielded 427 articles, of which 29 duplicates were removed, leaving 398 articles for title and abstract screening. Following abstract screening, 317 articles were excluded. An additional 57 articles were excluded after full-text review because they did not meet the predefined eligibility criteria, including criteria related to adolescent age, study setting, and reporting of results for ALWH when results were combined with other population groups. Ultimately, 24 articles met the inclusion criteria and were included in the scoping review.

Two reviewers independently screened all identified articles for eligibility using Covidence, with discrepancies resolved through discussion and consensus among the reviewers. Inter-rater reliability was high (Cohen’s κ = 0.73), reflecting substantial agreement between reviewers. The final set of 24 studies was confirmed based on the predefined inclusion and exclusion criteria. The PRISMA-ScR flowchart below (Fig 1) shows the results of the search and how the articles were screened [34,35].

**Fig 1:**
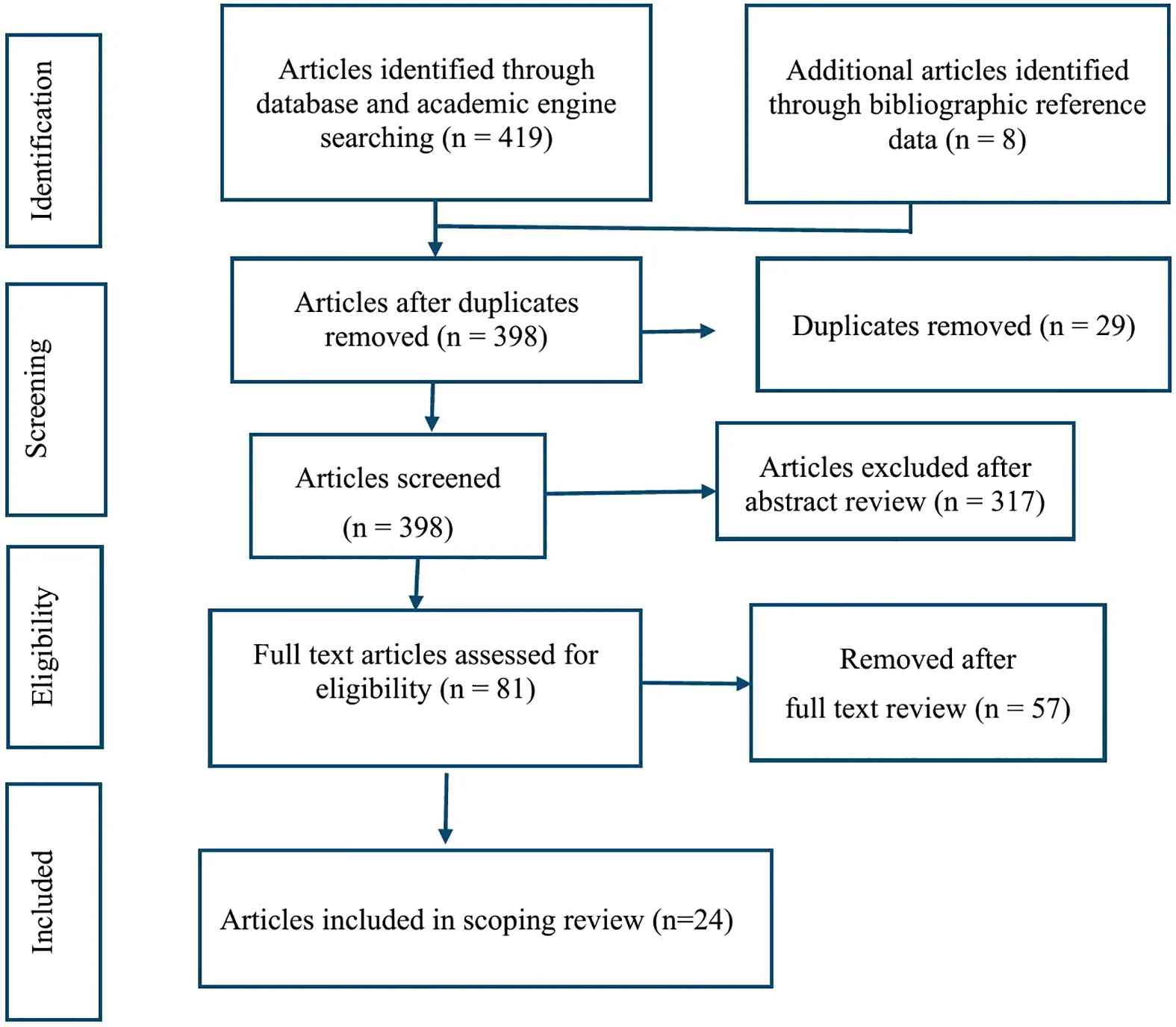
PRISMA-ScR flow diagram of study selection for the scoping review.

### Data extraction and analysis

Both authors participated in the data extraction process. Data were abstracted using an adapted Covidence template (Extraction 2). Data were extracted on the following characteristics: first author’s name, title, year of publication, study location, study design, sample size, participant characteristics, inclusion and exclusion criteria, and key study findings [36]. The extracted data were organized into a summary table for easier comparison.

Each author read and re-read the selected articles to gain a clear understanding of their content (content analysis). Both authors reviewed the extracted findings and identified specific mental health problems reported across the studies and reached a consensus on the grouping of similar mental health problems (categorization). This process culminated in descriptive categories of the common mental health problems. This collaborative approach was adopted to minimize errors during the review, particularly in the data extraction process [37]. Finally, the authors critically synthesized and summarized the findings of each study and then compared results across studies that examined mental health problems among adolescents living with HIV in SSA [36,38–41]. Due to the heterogeneity of study designs and findings among the included articles, this scoping review utilized descriptive content analysis and narrative synthesis [38–42]. Deductive and inductive content analysis was used to map and characterize the breadth of evidence on common mental health problems experienced by ALWH.

The Preferred Reporting Items for Systematic Reviews and Meta-Analyses extension for Scoping Reviews (PRISMA-ScR) [43] was used to guide the review (please see S1 PRISMA Checklist). All sources used in this review have been appropriately cited in the text and listed in the references section. A protocol was not registered for this scoping review.

## Results

### Characteristics of included studies

The 24 studies included in this review were conducted across the major regions of sub-Saharan Africa, namely Southern, Eastern, Western, and Central Africa. However, the geographical distribution of studies was uneven, and the included countries may not be fully representative of their respective regions. West Africa was represented by Nigeria, which contributed four studies, while Central Africa was represented by Cameroon, with one study. Southern Africa contributed the largest number of studies, with South Africa accounting for five studies and Botswana, Malawi, and Zimbabwe contributing two studies each, while Mozambique contributed one study. In East Africa, Uganda contributed four studies, whereas Kenya, Rwanda, and Tanzania each contributed one study. This uneven geographical distribution highlights the concentration of evidence in a limited number of countries and underscores the underrepresentation of many SSA countries in the available literature.

The characteristics of the studies included in this scoping review are further summarized in Table 1. Factors associated with common mental health problems were reported across multiple domains. Sociodemographic and developmental factors included female sex, school grade repetition, pregnancy, and age-related developmental circumstances. Psychosocial factors included HIV-related stigma and discrimination, negative life events, adverse childhood experiences, and physical or sexual abuse. HIV-related and clinical factors included frequent hospital admissions, mental health comorbidity, and unsuppressed viral load. Treatment-related factors included poor ART adherence. Across the 24 included studies, stigma and psychosocial adversity were repeatedly linked to depression, generalized anxiety, suicidality, and broader mental health difficulties. Additionally, mental health problems were also linked to poorer ART adherence and virological outcomes. However, the heterogeneity of study designs and measures limits direct comparison of effect sizes and prevents identification of a single consistently dominant correlate across all mental health outcomes.

**Table 1:** Characteristics of studies included in the scoping review on common mental health problems among adolescents living with HIV in sub-Saharan Africa (2020-2026).

Author, year	Study aim	Study design	Setting/ Sample	Prevalence *(& Comparator if present)*	Associated factors	Assessment tool
Adeyemo, et al., 2020	To determine the prevalence and correlates of depression and suicidality in ALWH	Cross-sectional study	Nigeria; n = 201, female and male ALWH aged 10–19 years	Current major depressive episodes: 16.9% Lifetime major depressive episodes: 44.8% Suicidality: 35.3%	Risk factors: Female gender, ACE, poor social support, physical abuse & HIV infection after birth	MINI-Kid
Akadri et al., 2024	To assess the prevalence of mental health distress and associated factors among ALWH	Cross-sectional study	Nigeria; n = 212, female and male ALWH aged 15–19 years	Mental health distress: 34.9%.	Risk factor: Alcohol consumption	GHQ-12
Birungi et al, 2021	To investigate alcohol and substance use (ASU) among ALWH in the sub-Saharan African setting of Uganda	Cross-sectional study	Uganda; n = 479, female and male ALWH aged 12–17 years	ASU: 5.9%, Alcohol: 4.3% Marijuana: 2.1%	Risk factors: Urban residence, caregiver psychological distress & PTSD	CASI-5
Buckley et al., 2020	To investigate the prevalence of mental illness in PHIV+ and HIV-uninfected adolescents in Soweto	Case-control study	South Africa; n = 81, female and male ALWH aged 13–19 years, and 81 without HIV of the same age group	Suicidality: 42% among ALWH vs. 28% for those without HIV. PTSD: 22% Eating disorders: 7% HIV + vs. 4% HIV- Substance abuse: 5% Mental health disorders: 58% HIV + vs. 41% HIV -	Risk factors: Female gender, HIV-, repeating a grade at school & physical and/or sexual abuse	PHQ-A C-PTSD-C MACI
Faidas et al., 2024	To describe stigma faced by ALWH and comorbid depression and its implications for future interventions	Cross-sectional study	Malawi; n = 25 female and male ALWH aged 13–19 with depression	Depressive symptoms: 24.7%	Risk factors: Fear, lack of awareness, stereotypes & prejudice about living with HIV	BDI-II
Gantsho et al., 2024	To determine the prevalence of depressive symptoms among ALWH	Cross-sectional study	South Africa; n = 125, female and male ALWH aged 10–19 years	Depressive symptoms: 44.8% Suicidal behaviours: 25.6%	Risk factors: School/home difficulties & previous suicide attempt	PHQ-A
Haas et al., 2020	To examine screening outcomes, associated patient characteristics, and the odds of unsuppressed viral load in ALWH screening positive for mental disorders	A cohort study	South Africa; n = 1,088, female and male ALWH aged 10–19 years	Depression: 4.4% Suicidality: 2.8% Anxiety: 2.2% PTSD: 3.2% Substance use issues: 1.7%	Protective factor: Female gender (substance abuse) Risk factors: Male gender (substance abuse), physical abuse & household conflicts	PHQ-9 AIP GAD-7 PC-PTSD-5 CAGE-AID
Kip et al., 2022	To assess the psychosocial challenges faced by ALWH attending adolescent-specific ART programs	A qualitative study	Malawi, n = 80, female and male ALWH aged 12–18 years	—	Risk factor: HIV-related stigma	—
Kwesiga et al., 2025	To estimate the prevalence and factors associated with mental health challenges among adolescents with unsuppressed viral loads in Northern Uganda	Cross-sectional study	Uganda; n = 121 female and male ALWH aged 10–18 years	Depression: 45.45% Emotional problems: 61.97%	Protective factors: Food & absence of recurrent infections (mental health challenges)	RCADS-25 PSRAT CAPS-CA
Mukangabire et al., 2021	To assess the prevalence and sociodemographic factors related to depression among ALWH	Cross-sectional research	Rwanda; n = 102 female and male ALWH aged 10–18 years	Depression: 31%	Risk factors: Not attending school & not living with biological parents (alive or deceased)	CES-DC
Musami et al., 2025	To determine the predictors and correlates of depression and suicidality among children and adolescents living with HIV and AIDS in Maiduguri, Nigeria	Cross-sectional study	Nigeria; n = 160 ALWH, female and male adolescents 10–18 years, female	Suicidality: 30.6% Depression:45.0%	Risk factors: Frequent hospital admissions & medication non-adherence	K-SADS-PL
Nanfuka et al., 2024	To explore the effects of anxiety, depression, and suicidal behaviour on the social functioning of ALHIV in a low-resource setting	Qualitative research	Uganda, n = 31 ALWH, female and male adolescents aged 10–19 years	Depression:12.9% Depression & suicidality: 12.9% Depression & anxiety: 45.2% Depression, anxiety & suicidality: 25.8% Anxiety: 3.2%	—	PHQ-A SCARED
Nanteza et al., 2025	To examine lifetime suicide attempts, depressive symptoms, and adverse experiences among adolescents with PHIV and demographically matched HIV-negative adolescents	Case-control study	Uganda; n = 50 ALWH, n = 50 without HIV, female, and male adolescents aged 12–19 years	Suicide attempts: 14% HIV + vs. 0% HIV -	Risk factors: Adverse childhood experiences (ACEs) and negative life events	PHQ-A ACEs questionnaire ALEQ
Ndongo et al., 2023	To estimate the prevalence and factors associated with depression in ALWH	Cross-sectional study	Cameroon; n = 302, female and male ALWH aged 10−19 years,	Depression: 26.5% suicidal ideation: 36.4% Anxiety: 29.1% for Low self-esteem:20.5%	Protective factors: Mother being alive (depression and suicidal ideation)	CDI MASC SEI
Nguyen et al., 2023	To examine mental health problems and their association with self-reported ART adherence among ALWH	Cross-sectional study	Mozambique; n = 213 female and male ALWH aged 15–19 years	Major depression: 11.7% Generalized anxiety disorder: 12.2% No PTSD	—	PHQ-9 GAD-7 CPSS-V
Njau et al., 2024	To explore the unmet mental health needs of ALWH to inform the development and implementation of a psychological intervention for depression in ALWH	Qualitative study	Tanzania; n = 45 participants composed of 15 HCPs, 15 both male and female ALWH aged 11–19 years old, and 15 caregivers.	—	Risk factors: Unmet mental health, poor academic performance & substance use	(no formal assessment)
Olashore et al., 2023	To determine the pattern of PDs in ALWH by comparing and exploring the differences in the pattern and their associated factors between CIAs and BIAs	Cross-sectional study	Botswana; n = 743, female and male ALWH aged 12–19 years	Depression: 23.6% Generalised anxiety disorder: 18.0% At least one psychiatric disorder: 52.9%	Risk factors: Male gender & unsuppressed VL *(CIAs)* Internalising disorders, females, poor counselling, and inability to accept HIV status *(BIAs)*	MINI-KID
Olashore et al., 2022	To estimate the prevalence of depression and suicidal behaviour and explore their associated factors	Cross-sectional study	Botswana; n = 622, female and male ALWH aged 12–19 years	Depression: 23% Suicidal behaviour: 18.9%	Risk factors: Female gender, loss of mother & unsuppressed VL (depression) Alcohol use, negative feelings about HIV status (suicidal behaviour) Protective factors: Social support & religious activities	MINI-KID
Ro et al., 2023	To determine the prevalence of depressive disorder and associated psychological factors among adolescents living with HIV/AIDS	Cross-sectional study	Nigeria; n = 105, female and male ALWH aged 10–19 years	Depressive disorder:14.3%.	Risk factors: HIV stigma, low self-esteem, female gender & romantic relationship	MINI-KID Internal HIV stigma scale Rosenberg self-esteem scale
Roberts et al., 2022	To identify the prevalence of probable common mental disorders among adolescent mothers and among adolescents experiencing the syndemic of motherhood and HIV	Cohort study	South Africa; n = 723 female ALWH and n = 467 female adolescents without HIV aged 10–19 years	Depressive symptoms: 12.7% adolescent mothers vs 5.9% for not Suicidality symptoms: 12.7% adolescent mothers vs 4.9% for not Anxiety symptoms: 3.6% adolescent mothers vs 1.0% for not Posttraumatic stress symptoms: 0% adolescent mothers vs 0.7% for not *Common mental disorder:* 23.0% for mothers living with HIV vs. 12.8% for those not mothers and without HIV	Risk factor: Being pregnant	MINI-KID RCMAS C-PTSD-C CDI-S
Simms et al., 2021	To identify factors associated with virological non-suppression among ALWH who had symptoms of common mental disorders	Cross-sectional study	Zimbabwe; n = 842, female and male ALWH aged 10–19 years	—	—	SSQ-14 PHQ-9 WHODAS 2.0
*Simms et al., 2022	To assess the effect of training Zvandiri peer counsellors known as CATS in PST on virological suppression and mental health outcomes	Cluster-randomised trial	Zimbabwe; n = 842, female and male ALWH aged 10–19 years	—	—	SSQ-14 PHQ-9 WHODAS 2.0
Wanjala et al., 2024	To investigate psychosocial and mental health challenges and the lived experiences of PHI adolescents in rural coastal Kenya	Qualitative study	Kenya; n = 40: 20 female and male ALWH aged 12–17 years and their 20 primary caregivers aged 32–56 years	—	Reported risk factors: Living in denial, HIV stigma (internalized & enacted), parental loss, limited parent support & unresponsive school system	—
Zanoni et al., 2021	To investigate modifiable factors to improve engagement in care before transition to adult care	Qualitative study	South Africa; n = 41 female and male ALWH aged 10–19 years, and 18 of their caregivers	—	Reported risk factors: HIV Denial, Unstable home environment, lack of social support, and learning difficulties	—

**Abbreviations:** Aces = Adverse Childhood Experiences, ALEQ = Adolescent Life Events Questionnaire, ALWH = adolescents living with HIV, ART = antiretroviral therapy, ASU = alcohol and substance use, BDI-II = Beck Depression Inventory-II, BIAs = behaviourally infected adolescents, CAGE-AID = Cut Down, Annoyed, Guilty, and Eye-Opener, Adapted to Include Drugs, CAPS-CA = Clinician-Administered Post-Traumatic Stress Disorder Scale for Children and Adolescents, CASI-5 = child and adolescent symptom inventory-5, CATS = Community Adolescent Treatment Supporters, CDI = Coopersmith Child Depression Inventory, CDI-S = Child Depression Inventory Short Form, CES-DC = Centre for Epidemiological Studies Depression Scale, CIAs = congenitally infected adolescents, CMD = common mental disorder, CPSS-V = Child PTSD Symptom Scale For DSM-5, C-PTSD-C = Child Post-Traumatic Stress Disorder, DSM-5 = Diagnostic and Statistical Manual of Mental Disorders, Fifth Edition, GAD-7 = Generalized Anxiety Disorder-7, GHQ-12 = General Health Questionnaire-12, HCPs = healthcare providers, HIV- = , HIV negative, K-SADS-PL = Schedule for Affective Disorders and Schizophrenia for School-Aged Children Present and Lifetime Version, MAC = Million Adolescent Clinical Inventory, MASC = Multidimensional Anxiety Scale for Children, MINI-Kid = Mini International Neuropsychiatric Interview for Children & Adolescents, PC-PTSD-5 = Primary Care PTSD Screen, PDs = psychiatric disorders, PHC = primary healthcare, PHI = perinatally-HIV infected, PHIV+ = HIV positive, PHQ-9 = Patient Health Questionnaire-9, PHQ-A = Patient Health Questionnaire for Adolescents, PST = problem-solving therapy, PTSD = post-traumatic stress disorder, RCADS-25 = Revised Child Anxiety and Depression Scale, RCMAS = Children’s Manifest Anxiety Scale-Revised, SCARED = Screen for Child Anxiety Related Disorder, SEI = Coopersmith Self-Esteem Inventory, SSQ-14 = Shona Symptom Questionnaire of CMD Symptoms, WHO = World Health Organization, WHODAS 2.0 = WHO Disability Assessment Schedule 2.0.

Note: * denotes a reused part of a dataset (Simms et al., 2022).

### Quality appraisal of included studies

The methodological quality and reporting of the included studies were assessed using established reporting and appraisal guidelines. Quantitative observational studies were evaluated using the STROBE (Strengthening the Reporting of Observational Studies in Epidemiology) checklist [44], while qualitative studies were assessed using the SRQR (Standards for Reporting Qualitative Research) guidelines [45]. Overall, the methodological quality of the 24 included studies was moderate. Most studies were cross-sectional, limiting causal inference. Many quantitative studies recruited participants from single HIV clinics or ART programs, which may have introduced selection bias by excluding adolescents not engaged in care. Qualitative studies used small purposive samples, limiting transferability. Most studies used standardized tools to assess depression, anxiety, suicidality, post-traumatic stress, and substance use. However, most of these tools were originally developed and validated in high-income settings, which may limit their applicability in sub-Saharan African contexts. Only four instruments have been validated in SSA specifically among adolescents and young people living with HIV: the Patient Health Questionnaire for Adolescents (PHQ-A) in Uganda (Cronbach’s α, 95% CI: 0.68–0.94) [46]; the Child PTSD Symptom Scale in South Africa (α = 0.84) [47]; the Children’s Depression Inventory–Short Version (CDI-S) in Malawi and Rwanda (α = 0.66) [48,49] and the Children’s Manifest Anxiety Scale-Revised (RCMAS) in South Africa (α = 0.85) [50]. These SSA-validated tools generally demonstrated acceptable to good internal consistency (α > 0.70) among HIV-affected adolescents, and youth, although contextual differences across SSA countries may still affect their broader applicability. Consistent with standard scoping review methodology, quality appraisal was conducted to characterize the body of evidence but was not applied as a criterion for study inclusion or exclusion.

### Prevalence of mental health problems

The 24 included studies demonstrated substantial variation in the prevalence of mental health problems among ALWH. This significant variation reflects differences in outcomes, measurement approaches, age groups, and study settings. Among studies reporting depressive outcomes, prevalence ranged from 14.3% for depressive disorder in Nigeria [51] to 45.5% for depressive symptoms in Uganda [52]. Current major depressive episodes were reported in 16.9% of ALWH in one Nigerian study, while lifetime major depressive episodes were reported in 44.8% [53]. A cohort study conducted in South Africa reported a 2.2% prevalence for anxiety, while one study done in Cameroon had a prevalence of 29.1% for anxiety [54].In Botswana, a prevalence of 18.0% for generalized anxiety disorder was reported [55]. Suicidality (e.g., suicidal ideation/thoughts, suicide plan/intent, suicide attempts) estimates ranged from 14.0% for suicide attempts in a Ugandan study [56] to 42% in a South African study [57]. In a case-control study done in South Africa, suicidality was higher among ALWH than HIV-negative adolescents (42% vs. 28%) [57]. Similarly, a case-control study in Uganda reported suicide attempts among 14.0% of ALWH compared with none (0%) among HIV-negative adolescents [56]. Alcohol and substance use (problem) was reported at 5.9% in Uganda [58], while a cohort in South Africa reported substance-use problems at 1.7% [59]. A prevalence of 3.2% for post-traumatic stress disorder (PTSD) was reported in South Africa [59]. In Uganda, a 62.0% prevalence of emotional problems was reported [52]. These estimates should be interpreted cautiously because the included studies used different outcomes and designs and therefore should not be treated as directly comparable.

### Spectrum of common mental health problems among ALWH in SSA

The scoping review identified six categories of common mental health problems among ALWH in SSA, which are described in detail below.

#### Category 1: Depressive disorders and symptoms.

Fourteen of the twenty-four reviewed studies reported the presence of depressive symptoms among ALWH. A study conducted by Akadri et al. [60] in Nigeria found that 34.9% of ALWH participants had depressive symptoms. The same study also revealed that approximately one-third (33%) of participants showed signs of mental health distress, particularly depression. Another Nigerian study reported a high prevalence of depressive symptoms among ALWH, with a current major depressive episode prevalence of 16.9% and a lifetime prevalence of 44.8% [53]. Overall, three studies conducted in different parts of Nigeria found depressive symptom prevalence ranging from 14.3% to 34.9% [51,53,60]. A study in Uganda reported a high prevalence of depression (45.5%) among 102 ALWH with anxiety symptoms. In contrast, 16.52% of ALWH experienced anxiety without comorbid depression [52].

Similarly, three other studies reported that ALWH were more susceptible to depressive symptoms [53,61–63]. These symptoms included persistent sadness, loss of interest in enjoyable activities, changes in sleep and appetite, fatigue, difficulty concentrating, and feelings of worthlessness or guilt. A study conducted by Haas et al. [59] in South Africa, further revealed that ALWH frequently experienced multiple mental health conditions. Notably, 39.6% of adolescents (19 out of 48) who screened positive for depressive symptoms in that study also showed signs of suicidality [59]. Depression and suicidality were commonly comorbid [59,64].

#### Category 2: Generalized anxiety.

Generalized anxiety symptoms were reported in ten out of the twenty-four studies as one of the most common mental health problems in ALWH. Roberts et al. [65] and Faidas et al. [61] concurred that anxiety among ALWH commonly manifested as persistent worry, fear of death, and general uneasiness. In Malawi, a study found that many ALWH internalized the widespread misconception that HIV is a death sentence [61]. This fear and worry were reinforced across various environments in school, home, and community settings. In schools, students were taught from a young age that HIV kills [61]. In keeping with the reported findings, 16.5% of ALWH in a study conducted in Uganda experienced anxiety [52].

#### Category 3: Suicidality.

Half of the 24 studies reviewed reported that ALWH experienced various forms of suicidality such as suicidal thoughts, suicide attempts, and self-harm [51,53,54,59,61,62,65–67]. In Nigeria, a study done by Adeyemo et al. [53] found that 35.3% of ALWH reported suicidal thoughts. Another Nigerian study revealed a suicidality prevalence of 42% among ALWH, compared to 28% in adolescents without HIV [68]. Similarly, Gantsho et al. [62] reported a suicidality prevalence of 25.6% in Johannesburg. Notably, some adolescents intentionally discontinued ART as a means of ending their lives, perceiving continued stigma and social rejection as worse than death [61]. Suicidal ideation was particularly prevalent among pregnant ALWH, who exhibited higher rates of suicidality than their non-pregnant counterparts [65]. These adolescents often expressed a desire to die, considered self-harm, or contemplated methods of suicide [57,65]. A case-control study in Uganda involving adolescents aged 12–19 years found a suicidality prevalence of 14.0% among ALWH, with no reported suicidality in the HIV-negative control group [56]. Another Ugandan study identified a suicide attempt prevalence of 25.8% [69]. Healthcare providers, ALWH, and their caregivers reported that unmet mental health needs exacerbated adolescents’ vulnerability to mental health problems, including suicidality [70].

#### Category 4: Emotional and behavioural problems.

According to four studies, ALWH experienced emotional and behavioural challenges related to the burden of living with an incurable and stigmatized virus [52,53,61,66]. In Uganda, emotional problems were reported among 61.97% of the 121 ALWH who participated in the study [45]. Many ALWH also experienced labile moods and difficulty controlling their emotions, resulting in affect that was perceived as unstable and unpredictable [48]. Recent studies conducted in Botswana and Zimbabwe have shown that adolescents living with HIV experience high rates of psychiatric disorders, particularly externalizing disorders such as conduct disorder and oppositional defiant disorder [55,66].

#### Category 5: Posttraumatic stress.

Posttraumatic stress signs and symptoms (easily startled, tense, irritable, detachment, nightmares, avoidance) were frequently observed among ALWH as a result of their traumatic experiences, such as public stigmatization, mocking, and discrimination [54,59,71]. Studies conducted in Cameroon, South Africa, and Mozambique demonstrated that ALWH often experienced distress, anxiety, and intrusive thoughts when recalling these events [54,59,71].

#### Category 6: Substance use.

Substance use, including alcohol and other drugs, was a coping mechanism adopted by some ALWH to manage the emotional toll of stigma and social isolation [59,61,62]. Some ALWH reported drinking alcohol more frequently or in greater quantities than intended to forget their mental health issues. Substance use was particularly prevalent among adolescents aged 15–19 years and was strongly associated with poor HIV-related health outcomes [59]. Similarly, a study conducted in Uganda demonstrated that alcohol and substance use (ASU) was common among ALWH, with a prevalence of 5.9%, with alcohol (4.3%) and marijuana (2.1%) being the most frequently reported. ASU was significantly associated with a psychiatric diagnosis of post-traumatic stress disorder [58].

## Discussion

This scoping review synthesized evidence on common mental health problems among adolescents living with HIV in sub-Saharan Africa. Consistent with existing literature, the findings show that ALWH commonly experience depressive disorder and symptoms, generalized anxiety, suicidality, emotional and behavioural problems, posttraumatic stress, and substance use problems [72].

Similar to previous reviews, depressive disorders and symptoms were very common [72,73]. Depressive disorders are more common among ALWH and can have profound consequences, including accelerated progression from HIV to AIDS [74,75]. Depressive disorders and symptoms are associated with increased viral load, and compromised immunity, resulting in greater vulnerability to opportunistic infections, poorer overall health outcomes, and a higher likelihood of dying [74,75]. These long-term risks highlight the critical need for routine mental health screening among ALWH. Mental health outcomes can be further improved by leveraging the successes of Teen Support Clubs through the integration of mental health services and screenings into all Teen Club Support Groups [26,76]. Healthcare providers working with ALWH should routinely screen for all forms of depressive disorders. Early and appropriate treatment is crucial to preserve the public health progress made through ART.

Consistent with previous reviews, our findings indicate that ALWH experience a broad range of mental health problems, including posttraumatic stress symptoms and emotional and behavioural difficulties [12,73]. These findings suggest that the psychosocial burden associated with living with HIV extends beyond depression and anxiety and may reflect the cumulative effects of HIV-related stigma, social adversity, and other potentially traumatic experiences. The observed vulnerability to posttraumatic stress and emotional and behavioural difficulties underscores the need for adolescent HIV services to incorporate comprehensive psychosocial assessment and support [77]. Integrating age-appropriate mental health screening, psychosocial support, and referral pathways into routine HIV care may help identify and address these needs earlier.

Consistent with the literature, generalized anxiety remains a key contributor to disability and illness worldwide [7]. This burden is amplified for ALWH, who face additional social anxiety linked to HIV-related stigma [75,78]. Daily stressors, including missed school, disrupted family responsibilities, and frequent clinic visits, contribute to this anxiety [75]. Fear of ART side effects further heightens anxiety symptoms [16,17]. Many ALWH also report increased anxiety during the transition from adolescent to adult HIV care, which can disrupt care engagement [78] and lead to outcomes similar to those seen with depression [1,79]. Because adolescence is a period of heightened vulnerability, untreated anxiety may impair reasoning and judgement, increasing the risk of unsafe sex and poor treatment adherence [31,80]. Routine screening for generalized anxiety and targeted psychological interventions is therefore essential for this population.

Compared with their peers, ALWH may experience a greater burden of suicidality, reflecting the cumulative effects of HIV-related stigma, social isolation, and other psychosocial challenges [81,82]. Strong social support may therefore serve as an important protective factor against suicidal thoughts and behaviours. The findings underscore the need for comprehensive, multi-level interventions that address the psychosocial and social determinants of suicidality among ALWH, including stigma and discrimination across family, school, community, and healthcare settings [83]. Such interventions may help strengthen social support, promote mental well-being, and reduce suicidality while supporting engagement in HIV care.

In line with existing evidence, ALWH experience alcohol and substance use problems and represent a highly vulnerable population requiring targeted clinical attention [12,72,73]. Conversely, a review by Evangeli et al. [84], which included studies from SSA and other settings, reported that most ALWH did not experience mental health or substance use problems. This difference may partly reflect the inclusion of studies conducted outside SSA, where social, cultural, and healthcare contexts may differ. The inconsistency highlights the need for region-specific research and the use of rigorous, culturally appropriate screening tools to accurately assess substance use problems among ALWH.

### Strengths and limitations of the review

The review’s findings should be interpreted with consideration of both its strengths and limitations. A key strength is the incorporation of systematic review elements, such as adherence to PRISMA-ScR guidelines, which helped lower bias and improve the scoping review’s accuracy and quality. Additionally, expert assistance from an Informationist at the Johns Hopkins Welch Medical Library in developing a comprehensive and systematic literature search strategy, along with the abstraction tool from Covidence, enhanced the rigor of the systematic literature search and review. However, several limitations should be noted. The geographic distribution of the included studies was heavily skewed: South Africa (5 studies) and Nigeria (4 studies) together accounted for nearly half of the evidence. Notably, Central Africa was represented by only one study from Cameroon. No Francophone sub-Saharan African country met the inclusion criteria. This imbalance was partly due to the English-language restriction. It limits the generalizability of the findings across the diverse health systems, languages, and HIV epidemic profiles in sub-Saharan Africa. Future reviews should include non-English and grey literature to address gaps in underrepresented regions, particularly Francophone West and Central Africa.

#### Significance and recommendations.

**Healthcare practice.** These findings highlight the need to integrate mental health screening and support into existing adolescent HIV services, including Teen Clubs. Brief, validated screening tools could be incorporated into Teen Club sessions and routine care visits, with trained peer supporters, counsellors, and other HIV-care providers conducting screening and offering basic psychosocial support within their scope. Strengthening peer-support models by training adolescent peer supporters to recognize warning signs and refer adolescents to trained providers, alongside equipping HIV-care providers to identify common mental health problems and follow clear referral pathways for specialized care, would support this integration. Task-shifted brief interventions, such as problem-solving therapy delivered under appropriate supervision, could further embed mental health support within existing HIV-care visits, reducing the burden on adolescents and caregivers.

**Health research.** These findings lay a foundation for future research on the relationship between HIV and mental health among ALWH in sub-Saharan Africa. Future research should prioritize longitudinal studies to examine how mental health problems develop over time and affect HIV treatment outcomes, and intervention studies testing integrated HIV–mental health services, including peer-delivered and task-shifted models. Research should also prioritize underrepresented settings, particularly Central and Francophone sub-Saharan Africa, and address substance use, posttraumatic stress, and emotional and behavioural symptoms; outcomes that remain understudied relative to depression among ALWH. Mixed-methods studies validating culturally adapted screening tools and identifying resilience factors that reduce suicidality amid HIV-related stigma are also needed.

**Health policy.** At the policy level, the findings support greater prioritization of mental health within adolescent HIV programs. Policies and program guidelines should facilitate the routine integration of mental health screening, basic psychosocial support, referral systems, and evidence-based interventions into HIV services. Investment in training HIV-care providers and peer supporters, strengthening referral networks, and ensuring access to specialized mental health professionals will be important for sustainable implementation. Such integration can support a more comprehensive and holistic approach to improving the health and well-being of ALWH across SSA.

## Conclusion

Adolescents living with HIV often experience mental health problems to a greater extent than their peers without HIV. Given the strong connection between HIV and mental health, especially among ALWH, it is crucial to strengthen efforts to address both health conditions simultaneously. There is a pressing need to incorporate routine mental health screening in all HIV care points, as research evidence demonstrates that addressing mental health problems is essential for achieving optimal HIV-related health outcomes. There is no health without mental health!

## Supporting information

S1 PRISMA Checklist PRISMA-ScR-Fillable-Checklist_10Sept2019. The PRISMA-ScR Fillable Checklist is an interactive 22-item reporting form designed to help researchers plan, complete, and accurately report transparent scoping reviews.
(DOCX)
