# Supplementary material for: Common mental health problems experienced by adolescents living with HIV in sub-Saharan Africa: A scoping review
Source: PLOS Glob Public Health. 2026 Oct 23;6(10):e0006832. doi: 10.1371/journal.pgph.0006832 (PMC13693942; doi:10.1371/journal.pgph.0006832)
Supplement: Supplementary file 2 [file pgph.0006832.s002.docx]

**PGPH-D-26-02316:** PLOS Global Public Health

Common mental health problems experienced by adolescents living with HIV in sub Saharan Africa: A scoping review

Overall, the paper addresses an important and timely topic: the range of mental health challenges experienced by adolescents living with HIV in sub-Saharan Africa.

The review appears to follow a clear scoping review approach, including use of PRISMA-ScR guidance, a broad search strategy, and thematic synthesis. These are important strengths. The manuscript also usefully highlights stigma and discrimination as central experiences that cut across many of the mental health problems described. However, the paper would be strengthened by providing more detail on the size of the problem, the factors linked to poor mental health, the quality of the studies included, and the practical implications for HIV services.

**Major comments**

1. The scoping review identifies themes but does not provide prevalence ranges or quantitative comparisons (e.g., between HIV-positive and HIV-negative adolescents).

Update table 2: Extract and summarize prevalence data for each mental health problem where available, similar to the systematic review's approach (Too et al., 2021) and where possible, compare rates with HIV-negative peers to contextualize the burden in the text.

2. The scoping review mentions associated factors in the discussion but does not present them In the results section or systematically analyze or categorize them to give context.

Systematically extract and categorize correlates (sociodemographic, psychosocial, HIV-related, adherence) for each mental health problem. Discuss which factors are most consistently associated and where evidence is lacking.

3. The scoping review does not assess the quality or risk of bias of included studies.

Incorporate a basic risk of bias or quality assessment (even if not as detailed as a systematic review), noting study design limitations, sample representativeness, and measurement validity and discuss how study quality may affect the interpretation of findings.

4. The review does not discuss the tools used to assess mental health or their cultural validity. Update table.

Summarize which screening or diagnostic tools were used across studies, and whether they were validated for the local context. Discuss implications for comparability and reliability of findings.

5. The review recommends more research but could be more specific.

Authors should Identify specific gaps (e.g., lack of longitudinal/interventional studies, underrepresentation of certain regions, limited focus on anxiety or PTSD). Suggest priority research questions and methodological improvements (e.g., need for mixed-methods, longitudinal designs, culturally adapted interventions).

6. The review recommends integration of mental health into HIV care but could provide more actionable guidance.

Offer concrete examples of how mental health screening and support can be integrated into existing HIV programs (e.g., Teen Clubs, peer support models). Discuss training needs for healthcare providers and potential models for task-shifting.

7. Authors note uneven geographical representation but does not analyze implications such as generalization and the need for research in underrepresented regions (e.g., Central Africa, Francophone countries).

**Minor comments:**

Table 2. There is only one table ….

In the themes, authors should remove the word ‘symptoms’ from the sub-headings (depressive symptoms, generalized anxiety symptoms, stigma and discrimination symptoms etc). And make this consistent throughout the document. Symptoms are different from mental disorder/mental health challenges.

3.1.4. Theme 4: Stigma and discrimination symptoms should be the last theme as it is not a direct mental disorder/illness, but a social determinants of health.

Lines 322-326: Authors can remove ‘other sub-Saharan African countries” and have the sentence read ”Recent studies conducted in Botswana and Zimbabwe, have shown that adolescents living with HIV experience high rates of psychiatric disorders, particularly externalizing disorders such as conduct disorder and oppositional defiant disorder.[46,57]”

With these revisions, the paper would provide a stronger and more useful contribution to the literature on adolescent HIV care and mental health.
