# Supplementary material for: Common mental health problems experienced by adolescents living with HIV in sub-Saharan Africa: A scoping review
Source: PLOS Glob Public Health. 2026 Oct 23;6(10):e0006832. doi: 10.1371/journal.pgph.0006832 (PMC13693942; doi:10.1371/journal.pgph.0006832)
Supplement: Supplementary file 3 [file pgph.0006832.s004.docx]

**PGPH-D-26-02316:** PLOS Global Public Health

Common mental health problems experienced by adolescents living with HIV in sub Saharan Africa: A scoping review

**Results**

- Make Supp Table 1, to be part of the main manuscript. This allows for the reader to quickly go through the table as they are reading the manuscript.
- Change the order of these subsections: Start with **Quality appraisal of included** studies followed by **Prevalence of mental health problems.** This situates the prevalence estimates better.
- Remove table 1 (Descriptive categories and supporting findings of common mental health problems among adolescents living with HIV in Sub-Saharan Africa), as the information is already in the text above.

**Discussion**

- Line 426-430 (Evidence suggests that social support can promote resilience and improve mental health outcomes among ALWH [78]. Therefore, HIV-specific support networks, such as Teen Clubs, should integrate psychosocial support alongside peer-to-peer support to strengthen resilience and promote mental health and well-being among ALWH.)….is out of place where it is. Can be included in the significance of the review/recommendation sections
- Combine significance of the review/recommendation sections into one coherent section.
