# Supplementary material for: Common mental health problems experienced by adolescents living with HIV in sub-Saharan Africa: A scoping review
Source: PLOS Glob Public Health. 2026 Oct 23;6(10):e0006832. doi: 10.1371/journal.pgph.0006832 (PMC13693942; doi:10.1371/journal.pgph.0006832)
Supplement: Supplementary file 4 [file pgph.0006832.s005.rtf]

Dear Prof. Seeley,
Thank you for giving us the opportunity to submit a revised draft of our manuscript, "Common mental health problems experienced by adolescents living with HIV in Sub-Saharan Africa: A scoping review," for publication in PLOS Global Public Health. We are grateful to you and the reviewers for the time and effort dedicated to evaluating our work, and for the insightful comments and constructive suggestions that have helped strengthen the manuscript.
We have carefully addressed each of the reviewers' comments and incorporated their suggestions throughout the manuscript. Corresponding changes are highlighted in yellow in the "Revised Manuscript with Track Changes" file. Below, we provide a point-by-point response to each reviewer's comment (also highlighted in yellow for ease of reference). Page and line numbers cited in our responses correspond to both the tracked-changes version and the clean revised manuscript.
We believe these revisions have substantially improved the manuscript, and we thank you and the reviewers again for your guidance.
Editor	
Comment	Author Response & Action Taken	Location in revised manuscript	
1. Please amend your online Financial Disclosure statement. If you did not receive any funding for this study, please simply state: "The authors received no specific funding for this work."	Thank you for this valuable feedback. The revised text reads as follows:
The authors received no specific funding for this work.	Pg. 24, Line 532
	
2. Please update your online Competing Interests statement. If you have no competing interests to declare, please state: "The authors have declared that no competing interests exist."	Thank you for pointing this out. The revised text reads as follows:
The authors received no specific funding for this work.	Pg. 24, Line 531

	
3. We note that your Data Availability Statement is currently as follows:
"Data available with the paper or supplementary information: All the data that support the underlying findings are included in the manuscript and its supplemental files. All data sources used in this review have been properly cited in the text and listed in the reference list."	Thank you for pointing this out. The revised text reads as follows:
All data sources (articles) used in this review have been properly cited in the text and listed in the reference list and Table 2.	Updated on the online portal	
4. Please provide separate main figure files in .tif or .eps format only and remove any figures embedded in your manuscript file. Please also ensure that all files are under our size limit of 10MB. Please leave the figure captions in the manuscript.	We were unsure whether a TIFF format of the PRISMA-ScR flow diagram was required in addition to its inclusion within the manuscript. We have now provided both and are happy to make any further adjustments as needed.	Pg. 23, Lines 208-224	
5. We have noticed that you have uploaded Supporting Information files, but you have not included a list of legends. Please add a full list of legends for your Supporting Information files before or after the references list.	Thank you for pointing this out. We have added the legends for the supporting documents:
S1 File: PRISMA-ScR-Fillable-Checklist_10Sept2019. The PRISMA-ScR Fillable Checklist is an interactive 22-item reporting form designed to help researchers plan, complete, and accurately report transparent scoping reviews. (DOCX)

S1 Table: Characteristics of studies included in the scoping review on common mental health problems among adolescents living with HIV in sub-Saharan Africa (2020-2026). Table 1 provides a comprehensive list of all studies included in the scoping review, organized in alphabetical order according to the surname of the first author. (DOCX)
	Pg. 25, Lines 544-555	

Reviewer 1	
Reviewer Comment	Author Response & Action Taken	Location in revised manuscript	
The review synthesizes a broader range of mental health problems (depression, anxiety, suicidality, stigma, emotional/behavioral problems, PTSD, substance use) among adolescents (10–19 years) living with HIV in SSA. Focuses on mapping the range of mental health issues, but lacks detailed risk of bias assessment and quantitative synthesis. Discusses the impact of mental health on ART adherence and health outcomes.	This is a great observation, thank you for pointing that out. We have addressed the comment as follows:
Detailed risk of bias assessment: “Quality appraisal of included studies
The methodological quality and reporting of the included studies were assessed using established reporting and appraisal guidelines. Quantitative observational studies were evaluated using the STROBE (Strengthening the Reporting of Observational Studies in Epidemiology) checklist [53], while qualitative studies were assessed using the SRQR (Standards for Reporting Qualitative Research) guidelines [54]. Overall, the methodological quality of the 24 included studies was moderate. Most studies were cross-sectional, limiting causal inference. Many quantitative studies recruited participants from single HIV clinics or ART programs, which may have introduced selection bias by excluding adolescents not engaged in care. Qualitative studies used small purposive samples, limiting transferability. Most studies used standardized tools to assess depression, anxiety, suicidality, post-traumatic stress, and substance use. However, most of these tools were originally developed and validated in high-income settings, which may limit their applicability in sub-Saharan African contexts. Only four instruments have been validated in SSA specifically among adolescents and young people living with HIV: the Patient Health Questionnaire for Adolescents (PHQ-A) in Uganda (Cronbach's á, 95% CI: 0.68–0.94) [55]; the Child PTSD Symptom Scale in South Africa (á = 0.84) [56]; the Children's Depression Inventory–Short Version (CDI-S) in Malawi and Rwanda (á = 0.66) [57, 58] and the Children's Manifest Anxiety Scale-Revised (RCMAS) in South Africa (á = 0.85) [59]. These SSA-validated tools generally demonstrated acceptable to good internal consistency (á > 0.70) among HIV-affected adolescents, and youth, although contextual differences across SSA countries may still affect their broader applicability. Consistent with standard scoping review methodology, quality appraisal was conducted to characterize the body of evidence but was not applied as a criterion for study inclusion or exclusion.”

Quantitative synthesis: “The 24 included studies demonstrated substantial variation in the prevalence of mental health problems among ALWH. This significant variation reflects differences in outcomes, measurement approaches, age groups, and study settings. Among studies reporting depressive outcomes, prevalence ranged from 14.3% for depressive disorder in Nigeria [44] to 45.5% for depressive symptoms in Uganda [45]. Current major depressive episodes were reported in 16.9% of ALWH in one Nigerian study, while lifetime major depressive episodes were reported in 44.8% [46]. A cohort study conducted in South Africa reported a 2.2% prevalence for anxiety, while one study done in Cameroon had a prevalence of 29.1% for anxiety [47].In Botswana, a prevalence of 18.0% for generalized anxiety disorder was reported [48]. Suicidality (e.g., suicidal ideation/thoughts, suicide plan/intent, suicide attempts) estimates ranged from 14.0% for suicide attempts in a Ugandan study [49]  to 42% in a South African study [50].  In a case-control study done in South Africa, suicidality was higher among ALWH than HIV-negative adolescents (42% vs. 28%) [50]. Similarly, a case-control study in Uganda reported suicide attempts among 14.0% of ALWH compared with none (0%) among HIV-negative adolescents [49].  Alcohol and substance use (problem) was reported at 5.9% in Uganda [51], while a cohort in South Africa reported substance-use problems at 1.7% [52].  A prevalence of 3.2% for post-traumatic stress disorder (PTSD) was reported in South Africa [52]. In Uganda, a 62.0% prevalence of emotional problems was reported [45]. These estimates should be interpreted cautiously because the included studies used different outcomes and designs and therefore should not be treated as directly comparable.”
	Pg. 14, Lines 297-321


Pg. 13, Lines 275-296	
The scoping review identifies themes but does not provide prevalence ranges or quantitative comparisons (e.g., between HIV-positive and HIV-negative adolescents). Update table 2: Extract and summarize prevalence data for each mental health problem where available, similar to the systematic review's approach (Too et al., 2021), and where possible, compare rates with HIV-negative peers to contextualize the burden in the text.
	We added a new Results subsection (Prevalence of mental health problems) and updated the table, now S1 Table (previously Table 2): The 24 included studies demonstrated substantial variation in the prevalence of mental health problems among ALWH. This significant variation reflects differences in outcomes, measurement approaches, age groups, and study settings. Among studies reporting depressive outcomes, prevalence ranged from 14.3% for depressive disorder in Nigeria [44] to 45.5% for depressive symptoms in Uganda [45]. Current major depressive episodes were reported in 16.9% of ALWH in one Nigerian study, while lifetime major depressive episodes were reported in 44.8% [46]. A cohort study conducted in South Africa reported a 2.2% prevalence for anxiety, while one study done in Cameroon had a prevalence of 29.1% for anxiety [47].In Botswana, a prevalence of 18.0% for generalized anxiety disorder was reported [48]. Suicidality (e.g., suicidal ideation/thoughts, suicide plan/intent, suicide attempts) estimates ranged from 14.0% for suicide attempts in a Ugandan study [49]  to 42% in a South African study [50].  In a case-control study done in South Africa, suicidality was higher among ALWH than HIV-negative adolescents (42% vs. 28%) [50]. Similarly, a case-control study in Uganda reported suicide attempts among 14.0% of ALWH compared with none (0%) among HIV-negative adolescents [49].  Alcohol and substance use (problem) was reported at 5.9% in Uganda [51], while a cohort in South Africa reported substance-use problems at 1.7% [52].  A prevalence of 3.2% for post-traumatic stress disorder (PTSD) was reported in South Africa [52]. In Uganda, a 62.0% prevalence of emotional problems was reported [45]. These estimates should be interpreted cautiously because the included studies used different outcomes and designs and therefore should not be treated as directly comparable.

Based on your suggestion, S1 Table (previously Table 2) has also been updated accordingly to reflect the prevalence and associated factors (where statistically significant) of each mental health problem when reported,
	Pg. 13, Lines 275-296	
Summarize which screening or diagnostic tools were used across studies, and whether they were validated for the local context. Discuss implications for comparability and reliability of findings.
	Thank you for this valuable feedback. The following changes have been made to address this comment (found under the appraisal of studies included…):
Most studies used standardized tools to assess depression, anxiety, suicidality, post-traumatic stress, and substance use. However, most of these tools were originally developed and validated in high-income settings, which may limit their applicability in sub-Saharan African contexts. Only four instruments have been validated in SSA specifically among adolescents and young people living with HIV: the Patient Health Questionnaire for Adolescents (PHQ-A) in Uganda (Cronbach's á, 95% CI: 0.68–0.94) [55]; the Child PTSD Symptom Scale in South Africa (á = 0.84) [56]; the Children's Depression Inventory–Short Version (CDI-S) in Malawi and Rwanda (á = 0.66) [57, 58] and the Children's Manifest Anxiety Scale-Revised (RCMAS) in South Africa (á = 0.85) [59]. These SSA-validated tools generally demonstrated acceptable to good internal consistency (á > 0.70) among HIV-affected adolescents, and youth, although contextual differences across SSA countries may still affect their broader applicability. Consistent with standard scoping review methodology, quality appraisal was conducted to characterize the body of evidence but was not applied as a criterion for study inclusion or exclusion.	Pg. 14, Lines 307-321	
The review recommends more research but could be more specific.
Authors should Identify specific gaps (e.g., lack of longitudinal/interventional studies, underrepresentation of certain regions, limited focus on anxiety or PTSD). Suggest priority research questions and methodological improvements (e.g., need for mixed-methods, longitudinal designs, culturally adapted interventions).
	We appreciate the reviewer's comment and have revised the relevant section to address this concern. The updated text now reads as follows:
Future research should address key gaps in the evidence base. Longitudinal studies are needed to examine how mental health problems develop and change over time and how they affect HIV treatment outcomes. Intervention studies should test integrated HIV–mental health services, including peer-delivered and task-shifted approaches. Research should prioritize underrepresented settings, particularly Central and Francophone sub-Saharan Africa, and give greater attention to substance use problems, posttraumatic stress and emotional and behavioral symptoms, which remain understudied relative to depression among ALWH. Mixed-methods studies are needed to validate culturally adapted screening tools for these problems and to identify protective and resilience factors that reduce suicidality among adolescents exposed to HIV-related stigma.
	Pg. 22-23, Lines 496-507	
The review recommends integration of mental health into HIV care but could provide more actionable guidance.
Offer concrete examples of how mental health screening and support can be integrated into existing HIV programs (e.g., Teen Clubs, peer support models). Discuss training needs for healthcare providers and potential models for task-shifting.
	Thank you for this valuable feedback. The following changes have been made to address this comment:
Mental health services should be integrated into existing adolescent HIV platforms. Brief, validated screening tools can be incorporated into Teen Club sessions and routine care visits, with trained peer supporters, counsellors, and other HIV-care providers conducting screening and offering basic psychosocial support. Adolescents who screen positive should have clear referral pathways to mental health professionals. Brief interventions, such as problem-solving therapy, can be delivered through task-shifting under appropriate supervision. Embedding these services within existing HIV-care visits may reduce the burden on adolescents and caregivers, while strengthening mental health support for ALWH across SSA.
	Pg. 23, Lines 508-517	
Authors note uneven geographical representation but does not analyze implications such as generalization and the need for research in underrepresented regions (e.g., Central Africa, Francophone countries).
	Thank you for this valuable feedback. The following changes have been made to address this comment:
The geographic distribution of the included studies was heavily skewed: South Africa (5 studies) and Nigeria (4 studies) together accounted for nearly half of the evidence. Notably, Central Africa was represented by only one study from Cameroon. No Francophone sub-Saharan African country met the inclusion criteria. This imbalance was partly due to the English-language restriction. It limits the generalizability of the findings across the diverse health systems, languages, and HIV epidemic profiles in sub-Saharan Africa. Future reviews should include non-English and grey literature to address gaps in underrepresented regions, particularly Francophone West and Central Africa.	Pg. 21-22, Lines 468-476	
The scoping review mentions associated factors in the discussion but does not present them In the results section or systematically analyze or categorize them to give context. Systematically extract and categorize correlates (sociodemographic, psychosocial, HIV-related, adherence) for each mental health problem. Discuss which factors are most consistently associated and where evidence is lacking.
	We appreciate the reviewer's comment and have revised the relevant section to address this concern. The updated text now reads as follows:
Factors associated with common mental health problems were reported across multiple domains. Sociodemographic and developmental factors included female sex, school grade repetition, pregnancy, and age-related developmental circumstances. Psychosocial factors included HIV-related stigma and discrimination, negative life events, adverse childhood experiences, and physical or sexual abuse. HIV-related and clinical factors included frequent hospital admissions, mental health comorbidity, and unsuppressed viral load. Treatment-related factors included poor ART adherence. Across the 24 included studies, stigma and psychosocial adversity were repeatedly linked to depression, generalized anxiety, suicidality, and broader mental health difficulties. Additionally, mental health problems were also linked to poorer ART adherence and virological outcomes. However, the heterogeneity of study designs and measures limits direct comparison of effect sizes and prevents identification of a single consistently dominant correlate across all mental health outcomes.	Pg. 12, Lines 261-274	
Reviewer 2	
Reviewer Comment	Author Response & Action Taken	Location in revised manuscript	
The review contributes to an important topic on how mental health influences uptake of ART and continuity of care for adolescents living with HIV.A few suggestions on how the manuscript could be improved: 
Introduction
 It would be nice to specify the adolescence age range being referred to.	We appreciate the reviewer's comment and have revised the relevant section to address this concern. The updated text now reads as follows:

This scoping review synthesized existing evidence on common mental health problems experienced by ALWH aged 10–19 years in sub-Saharan Africa (SSA).

The number of adolescents living with HIV (ALWH) continues to increase worldwide [1, 2]. Globally, an estimated 2.21 million adolescents aged 10–19 years were living with HIV in 2025, of whom 84% resided in sub-Saharan Africa (SSA) [2-4].	

Pg. 2, Lines 25-27


Pg. 4, Lines 63-64	
2. Starting the first paragraph with the statistics the authors have provided at the end could help give a general picture of the extent of the problem of adolescents living with HIV globally. The sentences at the beginning from line 34 to 39 could then help explain why focusing on adolescents is important.	Thank you for this valuable feedback; we agree with you. Here are the changes made (relocated sentences) in the first two paragraphs:
The number of adolescents living with HIV (ALWH) continues to increase worldwide [1, 2]. Globally, an estimated 2.21 million adolescents aged 10–19 years were living with HIV in 2025, of whom 84% resided in sub-Saharan Africa (SSA) [2-4]. A growing body of evidence indicates that ALWH are at increased risk of experiencing mental health problems and disorders [4, 5]. Globally, approximately one in seven adolescents experiences a mental health problem, which accounts for around 15% of the total disease burden among individuals aged 10–19 years [6]. In Africa, an estimated 37 million adolescents were living with a diagnosed mental health condition in 2019 [7]. Despite this substantial burden, mental health problems among adolescents frequently remain undiagnosed and untreated [7]. Untreated mental health conditions during adolescence can impair psychosocial development and limit individuals' ability to lead productive and fulfilling lives, with broader implications for societal and national development [7].

Adolescents account for approximately 23% of the population in SSA and play a critical role in the social and economic development of their families and communities, representing the next generation of the workforce and leadership [7, 8]. However, ALWH, particularly those in SSA, face substantial challenges that may undermine their health and future potential [8-11]. These challenges include limited access to healthcare, HIV-related stigma and discrimination, fear of disclosing HIV status to family members, orphanhood, caregiving responsibilities, poverty, and the ongoing impact of the HIV epidemic [8, 10]. The challenges associated with living with HIV may be particularly consequential during adolescence, a developmental period characterized by substantial physical, social, and cognitive changes as young people establish their identities and transition toward adulthood [20]. Adolescents also navigate evolving peer relationships and social connections, which can increase vulnerability to risky health behaviours and mental health problems [12, 13].  Although adolescence can be challenging in its own right, these challenges may be compounded among ALWH by having an incurable, potentially fatal, and frequently stigmatized virus [13]. Consequently, ALWH may experience substantial mental health challenges [14].
	Pg. 4, lines 63-92	
3. It would be great to expand the sentence beginning with "These challenges..." in line 39 - for example, what does family related difficulties mean?	Thank you for pointing this out. The revised text reads as follows: 
These challenges include limited access to healthcare, HIV-related stigma and discrimination, fear of disclosing HIV status to family members, orphanhood, caregiving responsibilities, poverty, and the ongoing impact of the HIV epidemic [8, 10].	Pg. 4, Lines 80-83	
4. The authors need to proof read the paper and ensure that all typographical and grammatical errors are corrected. For example, line 43 - 44 - ...one in seven adolescents experiences... Additionally, it would be great to avoid repeating Sub-Saharan Africa once it has been spelt out at the beginning. The same applies to adolescents living with HIV (ALWH).	Thank you for pointing this out. The revised text reads as follows:
Globally, approximately one in seven adolescents experiences a mental health problem, which accounts for around 15% of the total disease burden among individuals aged 10–19 years [6].	Pg. 4Lines 67-69	
5. Paragraph 3, I wondered if it would be better to specify that the issue is about ALWH. For example, line in line 54, would it be useful to include "among adolescents" after the word problems? The same with line 57 ... underdiagnosis of mental health issues among ALWH.	Thank you for pointing this out. The revised text reads as follows:
The mental health needs of ALWH may be further exacerbated by limitations within healthcare systems. Fragmented healthcare systems in which mental health and HIV services are not integrated can contribute to undiagnosed and untreated mental health problems [15, 16]. Moreover, limited mental health training and specialization among healthcare providers (HCPs) caring for ALWH [17, 18] may contribute to underdiagnosis and unmet mental health needs [19].   	Pg. 5, Lines 93-98	
Materials and methods
It would be great to justify why the specific databases were selected and why the period 2020 to 2026.	Thank you for this valuable feedback. The following changes have been made to address this comment:
The publication period was restricted to 2020–2026 to capture recent evidence reflecting contemporary HIV treatment, adolescent-friendly healthcare, and mental health service contexts, while providing an updated synthesis of the literature on mental health among ALWH in SSA.	Pg. 8, 176-179	
The search results are well presented. However, it would also be useful if you added details of how many people were involved in the screening process.	Thank you for pointing this out. The revised text reads as follows:
Two reviewers independently screened all identified articles for eligibility using Covidence, with discrepancies resolved through discussion and consensus among the reviewers. Inter-rater reliability was high (Cohen's ê = 0.73), reflecting substantial agreement between reviewers.	Pg. 9, Lines 193-196
