# Supplementary material for: Common mental health problems experienced by adolescents living with HIV in sub-Saharan Africa: A scoping review
Source: PLOS Glob Public Health. 2026 Oct 23;6(10):e0006832. doi: 10.1371/journal.pgph.0006832 (PMC13693942; doi:10.1371/journal.pgph.0006832)
Supplement: Supplementary file 5 [file pgph.0006832.s006.rtf]

Dear Prof. Seeley,
Thank you once for giving us another opportunity to submit a revised draft of our manuscript, "Common mental health problems experienced by adolescents living with HIV in Sub-Saharan Africa: A scoping review," for publication in PLOS Global Public Health. We are grateful to you and the reviewers for the time and effort dedicated to evaluating our work, and for the insightful comments and constructive suggestions that have helped strengthen the manuscript.
We have carefully addressed each of the reviewers' comments and incorporated their suggestions throughout the manuscript. Corresponding changes are highlighted in yellow in the "Revised Manuscript with Track Changes" file. Below, we provide a point-by-point response to each reviewer's comment (also highlighted in yellow for ease of reference). Page and line numbers cited in our responses correspond to both the tracked-changes version and the clean revised manuscript.
We believe these revisions have substantially improved the manuscript, and we thank you and the reviewers again for your guidance.
Editor	
Comment	Author Response & Action Taken	Location in revised manuscript	
Results

•	Make Supp Table 1, to be part of the main manuscript. This allows for the reader to quickly go through the table as they are reading the manuscript. 
•	Change the order of these subsections: Start with Quality appraisal of included studies followed by Prevalence of mental health problems. This situates the prevalence estimates better. 
•	Remove Table 1 (Descriptive categories and supporting findings of common mental health problems among adolescents living with HIV in Sub-Saharan Africa), as the information is already in the text above. 	This is a great observation; thank you for pointing that out. 

•	Based on your suggestion, S1 Table has been included within the manuscript
•	The revised sequence of the subsections is as follows: Quality appraisal of included studies followed by Prevalence of mental health problems.
•	Previously, Table 1(Descriptive categories and supporting findings of common mental health problems among adolescents living with HIV in Sub-Saharan Africa) was removed.	


•	Pg.15-19, Lines 325-329

•	Pg. 13, Line 277 (Quality appraisal of included studies)

•	Not application
	
Discussion
•	Line 426-430 (Evidence suggests that social support can promote resilience and improve mental health outcomes among ALWH [78]. Therefore, HIV-specific support networks, such as Teen Clubs, should integrate psychosocial support alongside peer-to-peer support to strengthen resilience and promote mental health and well-being among ALWH.) ….is out of place where it is. Can be included in the significance of the review/recommendation sections.


•	Combine significance of the review/recommendation sections into one coherent section. 	This is a great observation; thank you for pointing that out.
•	This has been removed: “Therefore, HIV-specific support networks, such as Teen Clubs, should integrate psychosocial support alongside peer-to-peer support to strengthen resilience and promote mental health and well-being among ALWH.”
•	Both sections have been merged as follows: Significance and recommendations
Healthcare practice. These findings highlight the need to integrate mental health screening and support into existing adolescent HIV services, including Teen Clubs. Brief, validated screening tools could be incorporated into Teen Club sessions and routine care visits, with trained peer supporters, counsellors, and other HIV-care providers conducting screening and offering basic psychosocial support within their scope. Strengthening peer-support models by training adolescent peer supporters to recognize warning signs and refer adolescents to trained providers, alongside equipping HIV-care providers to identify common mental health problems and follow clear referral pathways for specialized care, would support this integration. Task-shifted brief interventions, such as problem-solving therapy delivered under appropriate supervision, could further embed mental health support within existing HIV-care visits, reducing the burden on adolescents and caregivers.
Health research. These findings lay a foundation for future research on the relationship between HIV and mental health among ALWH in sub-Saharan Africa. Future research should prioritize longitudinal studies to examine how mental health problems develop over time and affect HIV treatment outcomes, and intervention studies testing integrated HIV–mental health services, including peer-delivered and task-shifted models. Research should also prioritize underrepresented settings, particularly Central and Francophone sub-Saharan Africa, and address substance use, posttraumatic stress, and emotional and behavioural symptoms; outcomes that remain understudied relative to depression among ALWH. Mixed-methods studies validating culturally adapted screening tools and identifying resilience factors that reduce suicidality amid HIV-related stigma are also needed.
Health policy. At the policy level, the findings support greater prioritization of mental health within adolescent HIV programs. Policies and program guidelines should facilitate the routine integration of mental health screening, basic psychosocial support, referral systems, and evidence-based interventions into HIV services. Investment in training HIV-care providers and peer supporters, strengthening referral networks, and ensuring access to specialized mental health professionals will be important for sustainable implementation. Such integration can support a more comprehensive and holistic approach to improving the health and well-being of ALWH across SSA.	


Pg. 27-28, Lines 515-546	


Reviewer 2	
Reviewer Comment	Author Response & Action Taken	Location in revised manuscript	
One point to clarify is on the PRISMA flow diagram. In the main text the authors mention that the initial search had 427 articles identified. However, the PRISMA flow diagram shows 419 articles identified through databases and search engines and 5 articles identified through bibliographic reference data. This makes the total number of articles 424 and not 427. It would also be useful to explain how they identified the bibliographic reference data in the search results section. At the moment it is not mentioned in the text - only in the PRISMA flow diagram.	This is a great observation; thank you for pointing that out. This has been rectified. Please see below:
Additionally, manual screening of the reference lists of included articles identified eight additional eligible studies, which were imported into Covidence and processed alongside records retrieved from the database search.
	Pg. 8, Lines 170-173
