# Supplementary material for: Common mental health problems experienced by adolescents living with HIV in sub-Saharan Africa: A scoping review
Source: PLOS Glob Public Health. 2026 Oct 23;6(10):e0006832. doi: 10.1371/journal.pgph.0006832 (PMC13693942; doi:10.1371/journal.pgph.0006832)
Supplement: Peer review history

# Peer review: Decision Letter 0

**DOI:** 10.1371/journal.pgph.0006832.r001

**Authors:** Janet Seeley

30 Jul 2026

PGPH-D-26-02316

Common mental health problems experienced by adolescents living with HIV in sub-Saharan Africa: A scoping review

PLOS Global Public Health

Dear Dr. Ndlangamandla,

Thank you for submitting your manuscript to PLOS Global Public Health. After careful consideration, we feel that it has merit but does not fully meet PLOS Global Public Health’s publication criteria as it currently stands. Therefore, we invite you to submit a revised version of the manuscript that addresses the points raised during the review process.

Both reviewers have given helpful suggestions for ways to revise this paper - which will strengthen this useful review.

Please submit your revised manuscript by Sep 28 2026 11:59PM. If you will need more time than this to complete your revisions, please reply to this message or contact the journal office at globalpubhealth@plos.org. When you're ready to submit your revision, log on to https://www.editorialmanager.com/pgph/ and select the 'Submissions Needing Revision' folder to locate your manuscript file.

Please include the following items when submitting your revised manuscript:

- A letter that responds to each point raised by the editor and reviewer(s). You should upload this letter as a separate file labeled 'Response to Reviewers'.
- A marked-up copy of your manuscript that highlights changes made to the original version. You should upload this as a separate file labeled 'Revised Manuscript with Track Changes'.
- An unmarked version of your revised paper without tracked changes. You should upload this as a separate file labeled 'Manuscript'.

Guidelines for resubmitting your figure files are available below the reviewer comments at the end of this letter.

As the corresponding author, your ORCID iD is verified in the submission system and will appear in the published article. PLOS supports the use of ORCID, and we encourage all coauthors to register for an ORCID iD and use it as well. Please encourage your coauthors to verify their ORCID iD within the submission system before final acceptance, as unverified ORCID iDs will not appear in the published article. *Only*  the individual author can complete the verification step; PLOS staff *cannot*  verify ORCID iDs on behalf of authors.

We look forward to receiving your revised manuscript.

Kind regards,

Janet Seeley

Academic Editor

PLOS Global Public Health

Journal Requirements:

1. Please amend your online Financial Disclosure statement. If you did not receive any funding for this study, please simply state: "The authors received no specific funding for this work."

2. Please update your online Competing Interests statement. If you have no competing interests to declare, please state: "The authors have declared that no competing interests exist."

3. We note that your Data Availability Statement is currently as follows:

"Data available with the paper or supplementary information:

• All the data that support the underlying findings are included in the manuscript and its supplemental files.

• All data sources used in this review have been properly cited in the text and listed in the reference list."

Please confirm at this time whether or not your submission contains all raw data required to replicate the results of your study. Authors must share the “minimal data set” for their submission. PLOS defines the minimal data set to consist of the data required to replicate all study findings reported in the article, as well as related metadata and methods (https://journals.plos.org/globalpublichealth/s/data-availability#loc-minimal-data-set-definition).

For example, authors should submit the following data:

- The values behind the means, standard deviations and other measures reported;

- The values used to build graphs;

- The points extracted from images for analysis.

Authors do not need to submit their entire data set if only a portion of the data was used in the reported study.

If your submission does not contain these data, please either upload them as Supporting Information files or deposit them to a stable, public repository and provide us with the relevant URLs, DOIs, or accession numbers. For a list of recommended repositories, please see https://journals.plos.org/globalpublichealth/s/recommended-repositories.

If there are ethical or legal restrictions on sharing a de-identified data set, please explain them in detail (e.g., data contain potentially sensitive information, data are owned by a third-party organization, etc.) and who has imposed them (e.g., an ethics committee). Please also provide contact information for a data access committee, ethics committee, or other institutional body to which data requests may be sent. If data are owned by a third party, please indicate how others may request data access.

4. Please provide separate main figure files in .tif or .eps format only and remove any figures embedded in your manuscript file. Please also ensure that all files are under our size limit of 10MB. Please leave the figure captions in the manuscript.

For more information about how to convert your figure files please see our guidelines: https://journals.plos.org/globalpublichealth/s/figures

5. We have noticed that you have uploaded Supporting Information files, but you have not included a list of legends. Please add a full list of legends for your Supporting Information files before or after the references list.

6. If the reviewer comments include a recommendation to cite specific previously published works, please review and evaluate these publications to determine whether they are relevant and should be cited. There is no requirement to cite these works unless the editor has indicated otherwise.

Additional Editor Comments (if provided):

[Note: HTML markup is below. Please do not edit.]

Reviewers' comments:

Reviewer’s Responses to Questions

**Comments to the Author**

1. Does this manuscript meet PLOS Global Public Health’s publication criteria?

Reviewer #1: Partly

Reviewer #2: Yes

**********

2. Has the statistical analysis been performed appropriately and rigorously?

Reviewer #1: N/A

Reviewer #2: N/A

**********

3. Have the authors made all data underlying the findings in their manuscript fully available (please refer to the Data Availability Statement at the start of the manuscript PDF file)?

The PLOS Data policy

Reviewer #1: Yes

Reviewer #2: Yes

**********

4. Is the manuscript presented in an intelligible fashion and written in standard English?

Reviewer #1: Yes

Reviewer #2: Yes

**********

Reviewer #1: The review synthesizes a broader range of mental health problems (depression, anxiety, suicidality, stigma, emotional/behavioral problems, PTSD, substance use) among adolescents (10–19 years) living with HIV in SSA. Focuses on mapping the range of mental health issues, but lacks detailed risk of bias assessment and quantitative synthesis. Discusses the impact of mental health on ART adherence and health outcomes.

Reviewer #2: The review contributes to an important topic on how mental health influences uptake of ART and continuity of care for adolescents living with HIV.

A few suggestions on how the manuscript could be improved:

Introduction -

1. It would be nice to specify the adolescence age range being referred to.

2. Starting the first paragraph with the statistics the authors have provided at the end could help give a general picture of the extent of the problem of adolescents living with HIV globally. The sentences at the beginning from line 34 to 39 could then help explain why focusing on adolescents is important.

3. It would be great to expand the sentence beginning with "These challenges..." in line 39 - for example, what does family related difficulties mean?

4. The authors need to proof read the paper and ensure that all typographical and grammatical errors are corrected. For example, line 43 - 44 - ...one in seven adolescents experiences... Additionally, it would be great to avoid repeating Sub-Saharan Africa once it has been spelt out at the beginning. The same applies to adolescents living with HIV (ALWH).

5. Paragraph 3, I wondered if it would be better to specify that the issue is about ALWH. For example, line in line 54, would it be useful to include "among adolescents" after the word problems? The same with line 57 ... underdiagnosis of mental health issues among ALWH.

Materials and methods

1. It would be great to justify why the specific databases were selected and why the period 2020 to 2026.

2. The search results are well presented. However, it would also be useful if you added details of how many people were involved in the screening process.

**********

what does this mean?). If published, this will include your full peer review and any attached files.

**Do you want your identity to be public for this peer review?**  If you choose “no”, your identity will remain anonymous but your review may still be made public.

For information about this choice, including consent withdrawal, please see our Privacy Policy

Reviewer #1: No

Reviewer #2: No

**********

[NOTE: If reviewer comments were submitted as an attachment file, they will be attached to this email and accessible via the submission site. Please log into your account, locate the manuscript record, and check for the action link "View Attachments". If this link does not appear, there are no attachment files.]

Attachment Submitted filename: PGPH-D-26-02316 - Peer review feedback.docx

# Author comment: Author response to Decision Letter 1

**DOI:** 10.1371/journal.pgph.0006832.r002

21 Aug 2026

Attachment Submitted filename: Response to Reviewers.rtf

# Peer review: Decision Letter 1

**DOI:** 10.1371/journal.pgph.0006832.r003

**Authors:** Janet Seeley

14 Sep 2026

PGPH-D-26-02316R1

Common mental health problems experienced by adolescents living with HIV in sub-Saharan Africa: A scoping review

PLOS Global Public Health

Dear Dr. Ndlangamandla,

Thank you for submitting your manuscript to PLOS Global Public Health. After careful consideration, we feel that it has merit but does not fully meet PLOS Global Public Health’s publication criteria as it currently stands. Therefore, we invite you to submit a revised version of the manuscript that addresses the points raised during the review process.

Thank you for the revisions to the manuscript - both reviewers have some additional small points for you to attend to which we would like you to address.

Please submit your revised manuscript by Oct 14 2026 11:59PM. If you will need more time than this to complete your revisions, please reply to this message or contact the journal office at globalpubhealth@plos.org. When you're ready to submit your revision, log on to https://www.editorialmanager.com/pgph/ and select the 'Submissions Needing Revision' folder to locate your manuscript file.

Please include the following items when submitting your revised manuscript:

- A letter that responds to each point raised by the editor and reviewer(s). You should upload this letter as a separate file labeled 'Response to Reviewers'.
- A marked-up copy of your manuscript that highlights changes made to the original version. You should upload this as a separate file labeled 'Revised Manuscript with Track Changes'.
- An unmarked version of your revised paper without tracked changes. You should upload this as a separate file labeled 'Manuscript'.

Guidelines for resubmitting your figure files are available below the reviewer comments at the end of this letter.

As the corresponding author, your ORCID iD is verified in the submission system and will appear in the published article. PLOS supports the use of ORCID, and we encourage all coauthors to register for an ORCID iD and use it as well. Please encourage your coauthors to verify their ORCID iD within the submission system before final acceptance, as unverified ORCID iDs will not appear in the published article. *Only*  the individual author can complete the verification step; PLOS staff *cannot*  verify ORCID iDs on behalf of authors.

We look forward to receiving your revised manuscript.

Kind regards,

Janet Seeley

Academic Editor

PLOS Global Public Health

**Journal Requirements:**

If the reviewer comments include a recommendation to cite specific previously published works, please review and evaluate these publications to determine whether they are relevant and should be cited. There is no requirement to cite these works unless the editor has indicated otherwise.

Please review your reference list to ensure that it is complete and correct. If you have cited papers that have been retracted, please include the rationale for doing so in the manuscript text, or remove these references and replace them with relevant current references. Any changes to the reference list should be mentioned in the rebuttal letter that accompanies your revised manuscript. If you need to cite a retracted article, indicate the article’s retracted status in the References list and also include a citation and full reference for the retraction notice.

**Additional Editor Comments (if provided):**

[Note: HTML markup is below. Please do not edit.]

Reviewers' comments:

Reviewer’s Responses to Questions

**Comments to the Author**

Reviewer #1: All comments have been addressed

Reviewer #2: All comments have been addressed

**********

publication criteria?

Reviewer #1: Yes

Reviewer #2: Yes

**********

3. Has the statistical analysis been performed appropriately and rigorously?

Reviewer #1: N/A

Reviewer #2: Yes

**********

4. Have the authors made all data underlying the findings in their manuscript fully available (please refer to the Data Availability Statement at the start of the manuscript PDF file)?

The PLOS Data policy

Reviewer #1: Yes

Reviewer #2: Yes

**********

5. Is the manuscript presented in an intelligible fashion and written in standard English?

Reviewer #1: Yes

Reviewer #2: Yes

**********

**Reviewer #1:**  Comments have been attended to.

**Reviewer #2:**  The manuscript has been significantly improved from the previous version and recommend it for publication as it contributes to an important topic in the literature. The authors have made good recommendations and acknowledged the limitations of the study.

One point to clarify is on the PRISMA flow diagram. In the main text the authors mention that the initial search had 427 articles identified. However, the PRISMA flow diagram shows 419 articles identified through databases and search engines and 5 articles identified through bibliographic reference data. This makes the total number of articles 424 and not 427. It would also be useful to explain how they identified the bibliographic reference data in the search results section. At the moment it is not mentioned in the text - only in the PRISMA flow diagram.

**********

what does this mean?). If published, this will include your full peer review and any attached files.

**Do you want your identity to be public for this peer review?**  If you choose “no”, your identity will remain anonymous but your review may still be made public.

For information about this choice, including consent withdrawal, please see our Privacy Policy

Reviewer #1: No

Reviewer #2: No

**********

[NOTE: If reviewer comments were submitted as an attachment file, they will be attached to this email and accessible via the submission site. Please log into your account, locate the manuscript record, and check for the action link "View Attachments". If this link does not appear, there are no attachment files.]

**Figure Resubmissions:**

Attachment Submitted filename: PGPH-D-26-02316 - Peer review feedback (1).docx

# Author comment: Author response to Decision Letter 2

**DOI:** 10.1371/journal.pgph.0006832.r004

28 Sep 2026

Attachment Submitted filename: Response_to_Reviewers_auresp_2.rtf

# Editor report: Decision Letter 2

**DOI:** 10.1371/journal.pgph.0006832.r005

**Authors:** Janet Seeley

5 Oct 2026

Common mental health problems experienced by adolescents living with HIV in sub-Saharan Africa: A scoping review

PGPH-D-26-02316R2

Dear Mr Ndlangamandla,

We are pleased to inform you that your manuscript 'Common mental health problems experienced by adolescents living with HIV in sub-Saharan Africa: A scoping review' has been provisionally accepted for publication in PLOS Global Public Health.

Before your manuscript can be formally accepted you will need to complete some formatting changes, which you will receive in a follow up email. A member of our team will be in touch with a set of requests.

Please note that your manuscript will not be scheduled for publication until you have made the required changes, so a swift response is appreciated.

IMPORTANT: The editorial review process is now complete. PLOS will only permit corrections to spelling, formatting or significant scientific errors from this point onwards. Requests for major changes, or any which affect the scientific understanding of your work, will cause delays to the publication date of your manuscript.

If your institution or institutions have a press office, please notify them about your upcoming paper to help maximize its impact. If they'll be preparing press materials, please inform our press team as soon as possible -- no later than 48 hours after receiving the formal acceptance. Your manuscript will remain under strict press embargo until 2 pm Eastern Time on the date of publication. For more information, please contact globalpubhealth@plos.org.

Thank you again for supporting Open Access publishing; we are looking forward to publishing your work in PLOS Global Public Health.

Best regards,

Janet Seeley

Academic Editor

PLOS Global Public Health

***********************************************************

Thank you for paying careful attention to the reviewer' comments.

Reviewer Comments (if any, and for reference):
